# OR6-004 – MRP8/14 promote MSU-crystal induced inflammation

**DOI:** 10.1186/1546-0096-11-S1-A99

**Published:** 2013-11-08

**Authors:** D Holzinger, N Nippe, T Vogl, K Marketon, V Mysore, T Weinhage, N Dalbeth, B Pool, T Merriman, D Baeten, A Ives, N Bagnoud, N Busso, D Foell, S Bas, C Gabay, J Roth

**Affiliations:** 1 Institute of Immunology, University Hospital Muenster, Muenster, Germany; 2 Department of Paediatric Rheumatology and Immunology, University Children’s Hospital Muenster, Muenster, Germany; 3 Department of Medicine, University of Auckland, Auckland, New Zealand; 4 Biochemistry Department, University of Otago, Otago, New Zealand; 5 Clinical Immunology and Rheumatology, University of Amsterdam, Amsterdam, the Netherlands; 6 Laboratoire de Rhumatologie, University of Lausanne, Lausanne, Switzerland; 7 Division of Rheumatology, University of Geneva, Geneva, Switzerland

## Introduction

Monosodium urate (MSU) crystal induced interleukin-1 (IL-1β) secretion is a critical pathogenic factor in the development of gout and serves as therapeutic target in these patients. Nevertheless, without co-stimulation by a pro-IL-1ß inducing factor, e.g. lipopolysaccharides (LPS), MSU alone cannot induce IL-1β secretion *in vitro*. The endogenous Toll-like receptor 4 (TLR-4) agonists myeloid related protein (MRP) 8 and MRP14 play a significant role in human inflammatory diseases, reflect disease activity and have been shown to be an important pathogenic factor in murine arthritis models.

## Objectives

To analyze the co-stimulatory properties of myeloid related protein-8 (MRP8) and MRP14 (endogenous Toll-like receptor 4 (TLR-4) agonists) in MSU crystal induced IL-1β secretion and their relevance in gout.

## Methods

The co-stimulatory effects of MRP8 and MRP14 on MSU-induced IL-1ß secretion were tested *in vitro* on primary human monocytes and macrophages as on murine macrophages and were confirmed by ELISA and Western Blot. Furthermore MSU induced release of MRPs from human neutrophils and monocytes were measured by ELISA. Impact of MRP was tested *in vivo* in a crystal-induced peritonitis model.

MRP8 and MRP14 was measured in paired serum and synovial fluid samples (n=15) and was detected in synovial tissue (n=10) of gout patients. Expression of MRPs was further correlated with disease activity in the serum of active and convalescent gout patients (each n=40)

## Results

MRP8 and MRP14 are released by MSU activated human neutrophils and monocytes and induce pro-IL-1ß production in monocytes. MSU induced IL-1β secretion is significantly increased by MRP co-stimulation in human and murine cells. Accordingly, targeted deletion of MRP14 in mice led to a significantly reduced response in MSU-induced inflammation *in vivo*. MRPs can be found in the synovia and synovial fluid of active gout patients and levels are significantly elevated compared to osteoarthritis patients. Moreover, the expression level of MRPs in serum of gout patients correlates positively with disease activity (mean ± 95% CI, active: 2020±420 ng/ml, convalescent: 920±70 ng/ml, controls: 430±100 ng/ml).

## Conclusion

MRP8 and MRP14 are endogenous enhancers of MSU crystal induced IL-1 β secretion by induction of pro-IL-1ß via TLR-4. The proteins can be found at the site of inflammation in active gout patients and their serum levels reflect disease activity in these patients. These findings indicate a new role of endogenous TLR-4 ligands in the pathogenesis of gout.

## Disclosure of interest

None declared

